# Retraction Note: Brain injury following cardiac arrest: pathophysiology for neurocritical care

**DOI:** 10.1186/s40560-016-0185-9

**Published:** 2016-09-20

**Authors:** Hiroyuki Uchino, Yukihiko Ogihara, Hidekimi Fukui, Miyuki Chijiiwa, Shusuke Sekine, Naomi Hara, Eskil Elmér

**Affiliations:** 1 Department of Anesthesiology, Tokyo Medical University, 6-7-1 Nishishinjuku, Shinjuku-ku, Tokyo, 160-0023 Japan; 2 Mitochondrial Pathophysiology Unit, Department of Clinical Sciences, Lund University, Box 117, 221 00 Lund, Sweden

## Retraction note

This article [1] has been retracted by the authors due to significant overlap with previous publications [2–4].
